# The Need for More Research and Public Health Interventions on Dengue Fever in Burkina Faso

**DOI:** 10.1371/journal.pntd.0002859

**Published:** 2014-06-19

**Authors:** Valéry Ridde, Mabel Carabali, Antarou Ly, Thomas Druetz, Seni Kouanda, Emmanuel Bonnet, Slim Haddad

**Affiliations:** 1 Montreal School of Public Health (ESPUM), Montreal, Canada; 2 University of Montreal Hospital Research Centre (CRCHUM), Montreal, Canada; 3 Dengue Vaccines Initiative, International Vaccines Institute, Seoul, South Korea; 4 Institut de Recherche en Sciences de la Santé, CNRST, Ouagadougou, Burkina Faso; 5 Identités et Différenciations de l'Environnement des Espaces et des Sociétés – Caen (IDEES), University of Caen Basse-Normandie, Caen, France; 6 Institut de Recherche pour le Développement (IRD), Paris, France; Centers for Disease Control and Prevention, United States of America

Since early November 2013, dengue fever has received considerable media attention in Burkina Faso [1], [2]. Nevertheless, scientific evidence, entomological knowledge, public information, health worker training, health system preparation, and public health interventions with regard to dengue fever in this country are sorely lacking. As such, our aim in this article is to draw attention to the need for rapid deployment of research and interventions on dengue fever in Burkina Faso, because it appears to have been overlooked, with the focus being, instead, on malaria.

This West African country, with a population of 15.75 million and where 100,000 children under five die each year—25,000 of them from malaria [3]—has been profoundly affected by that disease; malaria is today the primary cause of years of life lost and disability-adjusted life years [4]. However, a 2010 national survey revealed that only 54% of children under five with fever went to a health centre, and just 35% of all children received antimalarial drugs [5]. Nonetheless, long-lasting insecticide-treated mosquito nets (LLINs) were distributed nationally and at no charge in 2010 and 2013, and the sale of artemisinin-based combination therapies (ACTs) in health centres and by community health workers has been subsidized since 2010 [6], [7]. Rapid diagnostic tests (RDTs) for malaria were also introduced across the country in health centres in 2012 and by community health workers in villages in 2013, improving the detection of febrile diseases due to malaria. At the same time, the arrival of RDTs also made possible the identification of nonmalarial acute undifferentiated fever, including dengue. Given this, we believe it is becoming urgent to address this disease.

Dengue fever is a viral vector-borne disease caused by infection in humans of any of the four serotypes (DENV1, DENV2, DENV3, DENV4) of the dengue virus via the bite of the *Aedes* mosquito [8], [9]. In Burkina Faso, dengue represents an added burden in an epidemiological landscape dominated by malaria and in which national antimalarial programs are experiencing a number of problems in implementation (such as stock shortages of ACT and malaria RDTs) [6]. Despite this, neither the 2010 General Health Forum nor the March 2013 review of the health sector mentioned the problem of dengue fever.

It is because of these difficulties in the fight against malaria that the emergence of public and private debates around dengue is worrisome. The media are all focusing on this “new type” of fever, and both the general public and health workers are increasingly complaining that ACTs are ineffective against malaria [2], whereas, for instance, they may not actually be dealing with malaria, but dengue fever. In fact, it is not easy to make a differential diagnosis between dengue and other febrile diseases, such as malaria, in places where they coexist, given that they share many of the same symptoms, such as myalgia, arthralgia, and headache. The difficulties of differentiating among those diseases are compounded by the country's lack of diagnostic resources and laboratory facilities. Hence, at a press conference on another topic (LLINs) held on November 5, 2013, the Minister of Health was compelled to respond to numerous questions on dengue fever. He indicated that, between 2006 and 2008, there had been 683 confirmed cases of dengue in two locations: Ouagadougou, the capital city, and Nouna, a medium-sized city in the country's western region. In October 2013, the Ministry of Health rapidly implemented an investigation limited to four health centres in the capital. Of 111 rapid tests (SD BIOLINE Dengue Duo) performed on patients presenting acute febrile disease lasting two to seven days, 33 (29.7%) were positive, including 25 NS1 (25/33; 75.8%). The majority of these cases (90.9%) involved patients over 15 years of age. Laboratory analyses requested by the Ministry of Health are currently carried out in Dakar (Pasteur Institute) to confirm the dengue RDT results [10].

In fact, a major challenge relating to dengue is that very little is known about its presence in Burkina Faso, whether in humans or in the vector. The first epidemic of dengue appears to have occurred in 1925 [11], and other significant cases linked to serotype 2 were apparently identified in the 1980s [11], [12]. The fact remains that Burkina Faso is one of 34 African countries where cases have been reported since the 2000s [11], [13]. A study in 2003 of 191 blood donors and 492 pregnant women in a rural district and in the capital found seroprevalence ranging from 26.3% to 36.5%, but information about the serotype was not presented [14]. However, to our knowledge, no other study has been done in the ten years since then. There is thus no current estimate of the prevalence and incidence of infection in the population.

Our research team recently conducted, in 2013, a population survey in two of the country's medium-sized cities [15]. Of the febrile cases, in the city of Zorgho, 2.7% (3/112), and in the city of Kaya, 9.9% (15/151) showed positive IgM/IgG RTD results, including two AgNS1 positive cases (SD BIOLINE Dengue Duo). Laboratory analyses (reverse transcription PCR) of filter paper samples are being performed by Universidad del Valle (Cali, Colombia).

Additionally, although the role of the *Aedes aegypti* mosquito as the vector of dengue fever is well known [16], [17], and despite the fact that *A. aegypti* and *A. albopictus* are present in Burkina Faso [11], there has been no recent entomological study that would provide information about its distribution, especially in the country's large cities, where populations of this very urban mosquito have likely become widespread [2].

Given the resurgence of dengue fever in Burkina Faso and the current media attention, we believe action is urgently needed on two fronts: research and public health interventions (Box 1).

**Box 1. Needed Research and Actions on Dengue Fever in Burkina Faso**

Research:

- Population survey to measure prevalence and serotypes circulation
- Entomological studies on vector distribution and presence of infection

Public health interventions:

- Surveillance system and laboratory improvement
- Health staff training on diagnosis and management of dengue
- Vector control intervention
- Information targeting the general public

In terms of research, there is an urgent need for epidemiological studies, such as population surveys, to measure more precisely the burden of dengue fever, especially in large cities where the vector is very likely to be present. Information is needed not only on prevalence and incidence but also on the serotypes that are circulating, to understand the disease's behaviour and to facilitate the development of intervention strategies or training programs targeted to clinicians' needs [8]. The potential discovery of a new serotype could explain the occurrence of severe cases and previously unknown forms of the disease. This will require specific training of physicians to ensure treatment that is better adapted to the context. In addition, deaths and severe cases of the disease should be systematically investigated to uncover the determinants so that appropriate action can be taken. Entomological studies are also important to gather better information on vector distribution [18]. Up to now, very few researchers and research teams have tackled the problem of dengue fever in Burkina Faso. For example, the national yellow fever reference laboratory has no ELISA kits for performing analyses. While certain competencies are available, they have not been mobilized, because dengue fever had not been seen in a very long time and was not considered a public health problem. Until a disease is recognized as a problem, there will be no search for a solution to contain it [19]. Researchers and research funding agencies must therefore now mobilize to develop new knowledge.

The health system, which is already finding it very difficult to cope with malaria [20], is not prepared to contend with dengue fever. Health workers, mainly nurses and nurse assistants in both the public and private sectors, are not sufficiently trained for syndromic case management of dengue. Training based on the current WHO guidelines could be a starting point. A needs assessment should be done for health-worker training to improve the accurate diagnosis and treatment of patients with fever who do not have malaria. In general, the laboratory and surveillance systems need to be improved and decentralized. The population is not well informed on this subject, and a lot of erroneous information has been circulating since the media began focusing on this issue in early November 2013. In public health, rumours can very soon have disastrous effects [21]. Finally, the effort to control the vector, abandoned many years ago in Burkina Faso, must absolutely be revived, because it is one of the most effective means of dealing with the problem while waiting for a vaccine or another effective dengue control strategy [18]. The State and its technical and financial partners need to mobilize now so that the health system and its public health interventions can begin working on the most effective management of dengue fever [20].

## References

[1] Ministère de la santé (2013) Situation épidémiologique hebdomadaire. Semaine n°45 du 04 au 10/11/2013. Direction de la lutte contre la maladie. Ouagadougou: Ministère de la santé.

[2] Paco M (2013) Attention la Dengue est là ! L'Évenement. Ouagadogou. Available: http://www.evenement-bf.net/spip.php?article805. Accessed 22 May 2014.

[3] Lozano R, Naghavi M, Foreman K, Lim S, Shibuya K, et al (2012) Global and regional mortality from 235 causes of death for 20 age groups in 1990 and 2010: a systematic analysis for the Global Burden of Disease Study 2010. Lancet 380: 2095–2128. 23245604 10.1016/S0140-6736(12)61728-0 PMC10790329

[4] Institute for Health Metrics and Evaluation (2013) Global Burden of Diseases. Burkina Faso. Seattle: Institute for Health Metrics and Evaluation. Available: http://www.healthmetricsandevaluation.org/sites/default/files/country-profiles/GBD Country Report - Burkina Faso.pdf. Accessed 22 May 2014.

[5] INSD, Measure DHS, ICF Macro (2011) Enquête démographique et de santé et à indicateurs multiples (EDSBF-MICS IV), Rapport préliminaire, Burkina Faso, 2010. Ouagadougou: Institut national de la statistique et de la démographie. 40 p.

[6] Ridde V, Druetz T, Poppy S, Kouanda S, Haddad S (2013) Implementation fidelity of the national malaria control program in Burkina Faso. PLoS ONE 8: e69865. 23922831 10.1371/journal.pone.0069865 PMC3724672

[7] Zöllner C, De Allegri M, Louis VR, Yé M, Sié A, et al (2014) Insecticide-treated mosquito nets in rural Burkina Faso: assessment of coverage and equity in the wake of a universal distribution campaign. Health Policy Plan E-pub ahead of print. doi:10.1093/heapol/czt108 10.1093/heapol/czt108 24463333

[8] Rodriguez-Roche R, Gould EA (2013) Understanding the dengue viruses and progress towards their control. BioMed Res Int 2013: 690835. 23936833 10.1155/2013/690835 PMC3722981

[9] Bhatt S, Gething PW, Brady OJ, Messina JP, Farlow AW, et al (2013) The global distribution and burden of dengue. Nature 496: 504–507. 23563266 10.1038/nature12060 PMC3651993

[10] Ministère de la santé (2013) Rapport d'étape de l'investigation de cas suspects de Dengue dans la région sanitaire du Centre. Direction de la lutte contre la maladie. Ouagadougou: Ministère de la santé. 12 p.

[11] Amarasinghe A, Kuritsk JN, Letson GW, Margolis HS (2011) Dengue virus infection in Africa. Emerg Infect Dis 17: 1349–1354. 21801609 10.3201/eid1708.101515 PMC3381573

[12] Gonzalez J, Du Saussay C, Gautun J, McCormick J, Mouchet J (1985) La dengue au Burkina Faso (ex Haute-Volta)) : épidémies saisonnières en milieu urbain à Ouagadougou. Bull Soc Pathol Exot Filiales 78: 7–14. 3886182

[13] Were F (2012) The dengue situation in Africa. Paediatr Int Child Health 32 Suppl 1: 18–21. 22668445 10.1179/2046904712Z.00000000048 PMC3381440

[14] Collenberg E, Ouedraogo T, Ganamé J, Fickenscher H, Kynast-Wolf G, et al (2006) Seroprevalence of six different viruses among pregnant women and blood donors in rural and urban Burkina Faso: a comparative analysis. J Med Virol 78: 683–692. 16555290 10.1002/jmv.20593

[15] Kouanda S, Bado A, Yaméogo M, Nitièma J, Yaméogo G, et al (2013) The Kaya HDSS, Burkina Faso: a platform for epidemiological studies and health programme evaluation. Int J Epidemiol 42: 741–749. 23918849 10.1093/ije/dyt076

[16] Murray NEA, Quam MB, Wilder-Smith A (2013) Epidemiology of dengue: past, present and future prospects. Clin Epidemiol 5: 299–309. 23990732 10.2147/CLEP.S34440 PMC3753061

[17] Padmanabha H, Durham D, Correa F, Diuk-Wasser M, Galvani A (2012) The interactive roles of *Aedes aegypti* super-production and human density in dengue transmission. PLoS Negl Trop Dis 6: e1799. 22953017 10.1371/journal.pntd.0001799 PMC3429384

[18] Morrison AC, Zielinski-Gutierrez E, Scott TW, Rosenberg R (2008) Defining challenges and proposing solutions for control of the virus vector Aedes aegypti. PLoS Med 5: e68. 18351798 10.1371/journal.pmed.0050068 PMC2267811

[19] Rochefort DA, Cobb RW (1993) Problem definition, agenda access, and policy choice. Policy Stud J 21: 56–71.

[20] De Allegri M, Louis V, Tiendrébeogo J, Souares A, Yé M, et al (2013) Moving towards universal coverage with malaria control interventions: achievements and challenges in rural Burkina Faso. Int J Health Plann Manage 28: 102–121. 22689390 10.1002/hpm.2116

[21] Ghinai I, Willott C, Dadari I, Larson HJ (2013) Listening to the rumours: What the northern Nigeria polio vaccine boycott can tell us ten years on. Glob Public Health 8: 1138–1150. 24294986 10.1080/17441692.2013.859720 PMC4098042

